# Use of dietary fibers in enteral nutrition of critically ill patients: a systematic review

**DOI:** 10.5935/0103-507X.20180050

**Published:** 2018

**Authors:** Audrey Machado dos Reis, Ana Valéria Fruchtenicht, Sérgio Henrique Loss, Luis Fernando Moreira

**Affiliations:** 1 Hospital de Clínicas de Porto Alegre, Universidade Federal do Rio Grande do Sul - Porto Alegre (RS), Brasil.

**Keywords:** Dietary fiber/metabolism, Enteral nutrition, Intensive care, Critical care, Critically ill

## Abstract

To meet the nutritional requirements of patients admitted to intensive care units, it is necessary to establish a diet schedule. Complications associated with enteral nutrition by tube feeding are not uncommon and may reduce the delivery of required nutrient to patients in intensive care units. Research on the osmolality, fat content, caloric intensity and fiber content of formulas are under way, and a substantial number of studies have focused on fiber content tolerability or symptom reduction. We conducted a systematic review of dietary fiber use and safety in critically ill patients in 8 studies based on diarrhea, other gastrointestinal symptoms (abdominal distension, gastric residual volume, vomiting and constipation), intestinal microbiota, length of stay in the intensive care unit and death. We discussed the results reported in the scientific literature and current recommendations. This contemporary approach demonstrated that the use of soluble fiber in all hemodynamically stable, critically ill patients is safe and should be considered beneficial for reducing the incidence of diarrhea in this population.

## INTRODUCTION

Enteral nutrition (EN) is the preferred route for the nutritional support of critically ill patients under intensive care. These patients typically demand increased nutrients and energy as a result of catabolic stress;^(1)^ thus, adequate nutrition is crucial for these patients.^(2)^ To meet the nutritional requirements of patients admitted to intensive care units (ICU), it is necessary to establish a diet plan using enteral formulas as early as tolerated.^(3)^

Complications associated with EN via tube feeding are not uncommon,^(4)^ with diarrhea considered a major sign of intolerance.^(3-5)^ The metabolic activity of the luminal microbiota may be disrupted, thus affecting colonization resistance and contributing to complications.^(3)^ Consequently, EN formulations that have positive effects on gut ecology and intestinal function and provide appropriate nutritional support for ICU patients are of major interest.^(3)^ A substantial number of studies have focused on fiber content tolerability or symptom reduction.^(3)^ There is ample evidence of the beneficial effects^(6)^ of fiber enriched enteral formulas, which can stimulate the growth of beneficial normal flora bacteria, thereby inhibiting harmful bacteria.

Thus, this systematic review aims to identify the advantages and complications in association with the use of dietary fibers in critically ill patients.

## METHODS

A systematic literature search was conducted in accordance with the Preferred Reporting Items for Systematic Reviews and Meta-Analyses (PRISMA) statement.^(7)^ The search was performed in three databases: US National Library of Medicine and National Institutes of Health (PubMed), Latin American and Caribbean Health Sciences Literature (LILACS) and Scientific Electronic Library Online (SciELO). The search strategies for these databases were defined by terms related to dietary fiber ["dietary fiber", "dietary fiber-free", "dietary fiber-enriched", "dietary fiber-containing"] and critical care ["ICU", "intensive care", "critically ill", "life-threatening patients"]. Reviews, abstracts, dissertations, and case reports or articles published with more than a 15-year interval were excluded from this research.

Moreover, for inclusion in the review, studies needed to (1) specifically evaluate the use of dietary fiber in critically ill patients; (2) be published between January 1^st^, 2001 and December 1^st^, 2016; and (3) have been published in English, Spanish or Portuguese. Both randomized clinical trials and observational studies were included.

**Figure 1:**
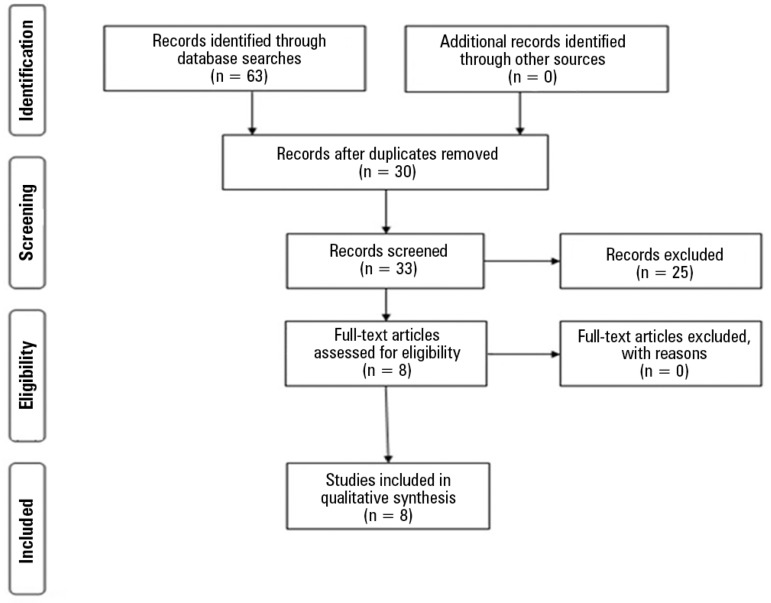
Flow chart of eligibility.

Finally, articles were screened according to the following steps: first, duplicates were excluded. The remaining articles were subsequently screened by title, abstract and text in full. Articles were selected based on the eligibility criteria as previously outlined. If eligibility could not be determined during the initial screening of the title and abstract, full-text articles were accessed to determine inclusion. Both study selection and data extraction were performed concurrently by two of the authors (AR and AV). In cases of doubt regarding the eligibility criteria, a third evaluator (LFM), who had also been engaged in the study, acted as a tiebreaker. PubMed, LILACS, and SciELO provided 61, 2, and 0 articles, respectively. Additional details are shown in figure 1.

## RESULTS

Of 63 studies, 8 studies (13%) were included in this review.^(2,3,6,8-12)^ Only one study (12.5%) evaluated children^(3)^ and no adults. Hemodynamic instability was considered an exclusion criterion.^(2,3,6,8-12)^ All articles involved dietary intervention exclusively by enteral tube feeding.

The durations of the protocols ranged from 4 to 7 days in 4 studies (50%)^(2,3,10,11)^ and from 2 to 5 weeks in the remaining studies.^(5,8,9,12)^ Three studies (37.5%) opted for supplementing the diet,^(6,9,10)^ while the other studies opted for the addition of fiber within the EN.^(2,3,8,11,12)^ The quality of fibers in the diet varied: 4 studies (50%) used mixed fiber types (soluble and insoluble)^(2,6,8,12)^ and 4 studies (50%) employed soluble fibers.^(3,9-11)^ One study used probiotics along with fiber.^(3)^ Lactobacillus paracasei and Bifidobacterium longum were used in their study,^(3)^ and the fiber amount ranged from 12.6 g/L to 12 g tid.

Additional details regarding these studies and the main results are provided in tables 1 and 2, respectively.

**Table 1:** Indexed articles used related to dietary fiber in critically ill patients

Author, country	Study	Sample	Sample exclusion criteria	Diet
Yagmurdur et al.,^(2)^ Turkey	Prospective, randomized	120 critically ill adults; cerebrovascular disease	Hemodynamic instability; sepsis; contraindications for enteral feeding; pancreatitis; gastrointestinal diseases; obesity; malnutrition syndrome; immunodeficiency; severe biochemical results on admission day; and patients who were given broad-spectrum antibiotics for a severe infection	Treatment group: diet with 15g/liter of mix fibers; Control group: Standard isocaloric and isonitrogenous diet
Simakachorn et al.,^(3)^ Thailand	Prospective, multicenter, randomized, double-blinded	94 critically ill children (1 - 3 years old) under mechanic ventilation	Enteral feeding contraindicated; recent surgery or other gastrointestinal disorders and immunodeficiency	Treatment group: diet with 2 probiotics and oligofructose/inulin fiber 2.6g/lL Control group: Standard isocaloric and isonitrogenous diet
O’Keefe et al.,^(6)^ United States	Clinical trial	13 critically ill adults; predominantly necrotizing pancreatitis (9 controls; 4 with diarrhea and ventilated patients - study group)	No exclusion criteria related	Both groups received mix fibers progressively up to maximum of 12g tid
Caparrós et al.,^(8)^ Spain	Multicenter, prospective, single-blinded	220 critically ill adults (122 cases; 98 controls)	APACHE II score < 8; MOD score > 5; pregnancy; terminals; cardiopulmonary resuscitation patients; diabetics; chronic gastrointestinal disease; renal or liver failure; cancer; immunodeficiency or previous use of corticosteroid salicylates; anti-inflammatory or immunosuppressive drugs	Treatment: 75g protein/liter, 11.8% arginine, 40% medium chain triglycerides; 8.9g mix fiber/liter Controls: Standard isocaloric, 62.5g protein/liter
Spapen et al.,^(9)^ Belgium	Prospective, randomized, double-blinded	25 adults; severe sepsis or septic chock	Terminal patients; inability to perform gastrointestinal treatment; pancreatitis; known diarrheal diseases or diarrhea up to 72 hours prior to inclusion; treatment modifying gastrointestinal transit; albuminemia; diabetes or any immunodeficiency	Treatment: diet supplemented with 22g of gum guar Controls: Standard isocaloric and isonitrogenous diet
Rushdi et al.,^(10)^ Egypt	Prospective, randomized	20 critically ill adults	Patients with short bowel syndrome; acute bacterial infection; enteral feeding contraindicated; sepsis or hyperthyroidism	Treatment: diet with 22g of gum guar/liter; Controls: Standard isocaloric and isonitrogenous diet
Spindler-Vesel et al.,^(11)^ Slovenia	Prospective, randomized	113 critically ill adults; trauma patients	No exclusion criteria stated	Group A: 4460mg arginine/liter; Group B: 22g guar gum/liter; Group C: standard diet; Group D: supplement of symbiotic 10^10^.
Chittawatanarat et al.,^(12)^ Thailand	Prospective, randomized, double-blinded	34 critically ill adults; surgical septic patients	Hemodynamic instability; enteral feeding contraindicated; pancreatitis; post-endoscopy < 24 hours bowel resection and anastomosis < 24 hours; gut diseases and enteric fistula	Treatment group: diet with 15.1g of mix fiber/liter; Control group: Standard isocaloric and isonitrogenous diet

APACHE II - Acute Physiology and Chronic Health Evaluation II; MOD - Multiple Organ Dysfunction.

**Table 2:** Indexed articles included and their main results

Author, country	Main results
Yagmurdur et al.,^(2)^ Turkey	The study group had less diarrhea than the control group (p < 0.001). The authors suggest that enteral nutrition should be initiated with fiber-enriched formulas rather than fiber-free formulas to avoid frequent feeding interruptions that cause protein energy malnutrition in intensive care unit patients
Simakachorn et al.,^(3)^ Thailand	The enteral formula enriched with soluble fiber and probiotic was well tolerated by critically ill children; it was safe and produced an increase in fecal bacterial groups of previously reported beneficial effects
O’Keefe et al.,^(6)^ United States	Fiber supplementation resulted in significant increases in fecal short chain fatty acids and microbial counts of specific butyrate producers, with a resolution of diarrhea in 3 of 4 patients. Thus, this supplementation has the potential to improve the microbiota mass and function, thereby reducing the risks of diarrhea as a result of dysbiosis
Caparrós et al.,^(8)^ Spain	Patients fed a diet enriched with soluble fiber had a significantly lower catheter-related sepsis rate than patients fed a standard high-protein diet. Patients fed the study diet for > 2 days showed a trend toward decreased mortality
Spapen et al.,^(9)^ Belgium	Enteral nutrition supplemented with soluble fiber is beneficial in reducing the incidence of diarrhea in tube-fed full-resuscitated and mechanically ventilated septic patients
Rushdi et al.,^(10)^ Egypt	Enteral nutrition fiber supplementation was related to a decrease of diarrheal episodes in intensive care unit patients with preexisting diarrhea and a trend towards lower plasma glucose and cholesterol levels
Spindler-Vesel et al.,^(11)^ Slovenia	The group that received soluble fiber and probiotic had significantly less combined infections (p = 0.003) and pneumonias (p = 0.03). Intestinal permeability decreased only in the symbiotic group (p < 0.05). Patients supplemented with symbiotic had lower intestinal permeability and fewer infections
Chittawatanarat et al.,^(12)^ Thailand	The fiber group had a lower mean diarrhea score (p = 0.005) and lower global diarrhea “score on the generalized scale (p = 0.005). In summary, a mixed fiber diet formula can reduce the diarrhea score in surgical, critically ill septic patients who received broad spectrum antibiotics

### Diarrhea

Spapen et al. found that the mean frequency of days with diarrhea was significantly lower in the fiber-treated group than in the controls (p < 0.001). They considered the total days of diarrhea (p < 0.01) or the number of cases that presented diarrhea for at least one day.^(9)^ Accordingly, Yagmurdur et al*.* also demonstrated a significant difference in diarrhea episodes that favored the fiber-enriched group (p < 0.001), which presented lower diarrhea scores in the last three days (p < 0.01), as well as diarrhea over the five days of the study (p < 0.001).^(2)^ Although it is not significant (p = 0.387), Simakachorn et al. found that diarrhea episodes were more frequent with standard formula.^(3)^ They also used probiotics in their study.^(3)^

Following a 14-day intervention, Chittawatanarat et al. found that the increase of the mean diarrhea scores was significantly lower in the fiber group than in the non-fiber group (p < 0.01).^(12)^ The fiber-receiving group presented a lower trend of incidence of patients with at least one day of diarrhea (p = 0.14). The overall incidence of diarrhea proportion per 100 patient-fed days in the mixed model was significantly lower in the fiber group (p = 0.01), even when nutrition started after patients had received broad-spectrum antibiotics (p = 0.04).^(12)^

The study with the largest number of cases was the only study that obtained results that did not favor the intervention, and the one in which the diet group had more diarrhea (p < 0.001).^(8)^

O'Keefe et al. administered mixed fibers to four patients in the study group who had diarrhea, which improved with progressive supplementation of 18g, 24g, and 35g/d in three patients.^(6)^ The other patient presented diarrhea even with a 36g/d fiber supplementation.^(6)^ In particular, this patient differed from the other patients as a result of the continuing need for broad-spectrum IV antibiotics (cefuroxime) and pantoprazole (PPI).^(6)^ Another study by Rushdi et al. that included patients who had diarrhea indicated a significant difference between liquid stools that favored the intervention group on the fourth day (p < 0.01).^(10)^

### Other gastrointestinal symptoms

Considering the gastric residual volume, Yagmurdur et al. did not identify differences between the groups.^(2)^ Moreover, only 4 patients had values that exceeded 500 mL per day, including 3 patients in the control group and 1 patient in the fiber-enriched diet group.^(2)^ In contrast, Caparrós et al. showed increased gastric residues in the diet intervention group (p < 0.001).^(8)^

Spindler-Vesel et al. conducted a study with four groups.^(11)^ The glutamine-supplemented group had a significantly lower gastric residual volume than the fiber-only group (p < 0.05), as well as the probiotic plus fiber-supplemented group (p < 0.02).^(11)^ Moreover, patients in the control group exhibited less gastric retention than patients in the probiotic plus fiber-supplemented group (p < 0.04).^(11)^ With respect to gastric empting,^(11)^ there was no difference among the group that received only fiber supplementation, the control group, and the probiotic and fiber supplementation group.

Three articles demonstrated that vomiting was less frequent in the fiber-supplemented group; however, there were no significant differences.^(2,3,10)^

Abdominal distension was described in some articles. O'Keefe et al. did not identify clinically relevant differences.^(6)^ However, abdominal distension was similar in both groups (p = 0.83) in Simakachorn et al.,^(3)^ while distension was less observed in the control group (30% *versus* 42%) in Yagmurdur et al.^(2)^

Constipation results varied. Caparrós et al. showed that controls had significantly more episodes of constipation (p < 0.005).^(8)^ Rushdi et al. identified only one patient among 4 cases with constipation in the control group (25%),^(10)^ and Yagmurdur et al. identified similar cases in both groups.^(2)^

### Intestinal microbiota

Only two articles included an intestinal microbiota analysis.^(3,6)^ Simakachorn et al. described a significant difference in the total bifidobacterial counts. Although decreased in controls (14 days, p = 0.046),^(3)^ the viable lactobacilli counts gradually increased during the study in both treatment groups.^(3)^ Subjects who received EN supplemented with symbiotics (prebiotics and probiotics) presented a trend for a larger population of lactobacilli than subjects who received non-supplemented formula (p = 0.085).^(3)^ Similar counts against baseline, on average, were low for both groups after 7 and 14 days, which suggests a relatively unstable microbiota.^(3)^ No differences were identified concerning bacterial diversity (number of bands) in both groups throughout the study.^(3)^

After comparing healthy subjects to patients with diarrhea, O'Keefe et al. determined that fecal short chain fatty acids (SCFAs) were significantly lower in patients with diarrhea (acetate: p < 0.012; propionate p < 0.007; and isobutyrate p = 0.35).^(6)^ The bacterial composition was strikingly different, with phyla comprising up to 35% and 60% of the microbiota for healthy subjects and patients,^(6)^ respectively. However, there was a 50 percent decrease in the amount of firmicutes, which contain the major butyrate-producers, in patients compared to a 30 percent decrease in controls.^(6)^ Furthermore, the proportions of phyla had a 97 percent reduction in the predominantly butyrate producers and starch degraders, at the genus level, from Clostridia clusters prior to fiber supplementation.^(6)^ After 2 to 5 weeks of fiber supplementation in diarrhea patients group, there was a 6-fold increase in firmicutes, followed by a significant increase in fecal SCFAs (acetate p = 0.01; propionate p = 0.006; and butyrate p = 0.04).^(6)^ Microbial counts, such as major butyrate producers, *E. rectale, E. hallii,* and *R. intestinalis*, which belong to the Clostridia cluster,^(6)^ also increased. Similarly, there were increases in *R. bromii, R. obeum,* and *Sporobacter terminitidis*, organisms that degrade starch and other complex carbohydrates.^(6)^

### Length of intensive care unit stay and death

Caparrós et al. found that the ICU stay was significantly shorter in the control group (p = 0.01).^(8)^ Nevertheless, Spapen H et al.^(9)^ stated that no difference was identified between the control and study groups. Chittawatanarat et al. showed significant differences between ICU and length of hospital stay (LOS) for the fiber supplemented group (p = 0.07).^(12)^

Three studies presented details regarding death. There was a lower number of in-hospital deaths in all studies; however, there were no statistically significant differences among the study groups.^(8,9,11)^ As expected, Spindler-Vesel et al. showed that mortality was significantly associated with a higher age (p < 0.0004), higher Acute Physiology And Chronic Health Evaluation II (APACHE II) score (p < 0.015), and higher Multiple Organ Failure (MOF) score (p < 0.02).^(11)^ Furthermore, less feeding in the first four days (p < 0.04) and higher gastric volume retention (p < 0.0004) were also associated with death.^(11)^ Caparrós et al. reported that mortality after 6 months was considerably different on cumulative survival curves favoring the intervention group (p < 0.05).^(8)^

## DISCUSSION

From a physiological point of view, dietary fiber may be divided in two groups: water soluble (e.g., pectin and β-glycan) and water insoluble (e.g., cellulose).^(13)^ Non-fermentable insoluble fibers increase the volume of stool, and because of mechanical stimulation of the gut mucosa, they decrease fecal transit time.^(14)^ Almost all soluble fiber fractions are completely fermented in the large bowel.^(14)^ During bacterial fermentation of soluble fiber, SCFAs, mainly butyrate, are produced.^(15)^ Butyrate is considered the main energy substrate for enterocytes and a stimulator of growth and differentiation.^(15)^ Moreover, SCFAs are crucial to inhibit pro-inflammatory mediator activities in the intestinal epithelium.^(16)^ Fibers promote beneficial bacterial growth, such as lactobacillus and bifidobacteria, which are referred to as prebiotics because they improve gut barrier function, host immunity, and reduce overgrowth of pathogenic bacteria, such as Clostridia.^(17)^ For this reason, fibers are considered an important anti-diarrheal tool.^(17)^

The frequency of diarrhea in EN patients ranged from 2% to 95%. This substantial range was a result of the distinct definitions of diarrhea and different measurement methods.^(18)^ In critically ill patients, this result ranged from 29% to 72%.^(19)^ Whelan et al. assumed that EN changes transit time and secretory mechanisms, thus contributing to worsen an already critical scenario.^(18)^ Yagmurdur et al. identified diarrhea as the most frequent complication, which occurred in half of the patients. The authors considered EN as a contributing factor to diarrhea in ICU patients because it changes gut physiology and gastrointestinal microbiota.^(2)^

No study addressing critically ill patients has been designed to consider only insoluble fibers. Studies typically consider insoluble and soluble fibers mixed together for this ICU population.^(20)^ Older studies, which have not been included in this review, have demonstrated contrasting results for the use of mixed fibers in the management of diarrhea in the ICU.^(21-23)^

Dobb and Towler demonstrated that diarrhea occurred more frequently in patients who were administered a soy-polysaccharide enriched diet.^(21)^ Frankenfield and Beyer showed that tube feedings containing soy-polysaccharide fiber did not seem to have an effect on bowel function in well-nourished head-injured patients.^(22)^ Guenter et al. reported that soy-polysaccharide fiber reduced the incidence percentage of diarrhea per total feeding days, as well as the frequency of positive Clostridia toxin, although it was not significant.^(23)^ These studies analyzed only one type of fiber, in addition to being conducted many years ago. Nevertheless, a meta-analysis performed in 2008, which included 13 studies, indicated that soluble fibers could significantly reduce episodes of diarrhea in patients (p = 0.03), but not in patients under intensive care.^(24)^ They reported that the beneficial mean fiber intake amount is approximately 30g/day in most studies.^(24)^ This study included both healthy individuals and hospitalized patients. In our findings, which considered critically ill patients, they received approximately 2.6g/L to 12g tid of fiber.

Studies that were not conducted inside an ICU showed benefits in dietary fiber use. Kurasawa et al. demonstrated that dietary fiber increases the stool weight and contributes to easier defecation.^(25)^ Salmerón et al. reported dietary fiber^(26)^ effects on the management of glucose. Fibers improve gut barrier function and host immunity, thus reducing the overgrowth of pathogenic bacteria, such as Clostridia.^(17)^ The immunological support provided by fructo-oligosaccharides includes increased T-lymphocytes in adults, an increased antibody response to vaccines in infants, and reduced antibiotic consumption.^(26-29)^ Majid et al. reported that fibers reduced the diarrhea incidence in patients receiving enteral nutrition.^(30)^

Enteral nutrition is a contributing factor to ICU diarrhea because it alters gut physiology.^(2)^ Whelan et al. suggested that enteral feeding changes the transit time, secretory mechanisms, and microbiota in the gastrointestinal tract.^(18)^ Therefore, diarrhea and a greater gastric residual volume were identified as the most frequent complications in this patient profile,^(31)^ although this analysis may widely vary because of the different gastric residual volume measurement methods. Moreover, being careful to increase diet infusion and the use of metoclopramide may be factors that affect gastric emptying and cause low gastric residual volumes.^(2)^

This review, which included only ICU patients, showed that diarrhea was improved in most studies.^(2,6,9,10,12)^ These findings demonstrate the importance of fiber use for critical care. In addition, studies indicated potential improvements in infections,^(8,11)^ as well as mortality,^(8)^ even if these effects are discrete.

The last publication of the European Society for Clinical Nutrition and Metabolism (ESPEN) in 2006 did not include recommendations for the issue.^(32)^ This finding is similar to the Canadian Society, which considered the published data were not sufficiently consistent to recommend the daily use of fibers in the ICU.^(20,33)^ However, in a recent publication, the American Society for Parenteral and Enteral Nutrition (ASPEN) recommended only soluble fiber for critically stable hemodynamic patients who developed diarrhea.^(34)^ Furthermore, the use of insoluble fiber for critically ill patients in general was contraindicated.^(34)^ Moreover, although the articles used for this recommendation were based on case reports,^(34)^ both fiber types should be avoided for patients at risk for mesenteric ischemia or severe motility impairment.

One should be aware that this systematic review presents several limitations. Although the whole protocol that includes all relevant articles was carefully applied, the small number of studies certainly hinders broader considerations. Moreover, some studies were conducted and published many years ago, which also hampers comparisons to current studies when more technologically processed diets and resources in the ICU have been developed.

## CONCLUSION

The use of soluble fiber in all hemodynamically stable, critically ill patients is safe and may be considered to be beneficial for reducing gastrointestinal symptoms, mainly diarrhea. Therefore, the use of soluble fiber may assist in the treatment of critically ill patients. Thus, more studies are needed to improve the routine use of an enriched fiber diet in intensive care unit patients.
